# Estimated Nutritive Value of Low-Price Model Lunch Sets Provided to Garment Workers in Cambodia

**DOI:** 10.3390/nu9070782

**Published:** 2017-07-21

**Authors:** Jan Makurat, Aarati Pillai, Frank T. Wieringa, Chhoun Chamnan, Michael B. Krawinkel

**Affiliations:** 1 Institute of Nutritional Sciences, Justus Liebig University Giessen, Wilhelmstrasse 20, 35392 Giessen, Germany; aaratipillai@gmail.com (A.P.); krawinkel@fb09.uni-giessen.de (M.B.K.); 2 UMR 204 Nutripass, Institut de Recherche pour le Développement (IRD), IRD/UM/SupAgro, 911 Avenue d’ Agropolis, 34394 Montpellier, France; franck.wieringa@ird.fr; 3 Department of Fisheries Post-Harvest Technologies and Quality Control (DFPTQ), Fisheries Administration, Ministry of Agriculture, Forestry and Fisheries (MAFF), 186 Preah Norodom Boulevard, 12000 Phnom Penh, Cambodia; chhounchamnan@gmail.com

**Keywords:** nutritive value, recommended dietary allowance, lunch provision, Cambodia, garment factory, staff canteen, underweight, anemia, micronutrient deficiency, malnutrition

## Abstract

Background: The establishment of staff canteens is expected to improve the nutritional situation of Cambodian garment workers. The objective of this study is to assess the nutritive value of low-price model lunch sets provided at a garment factory in Phnom Penh, Cambodia. Methods: Exemplary lunch sets were served to female workers through a temporary canteen at a garment factory in Phnom Penh. Dish samples were collected repeatedly to examine mean serving sizes of individual ingredients. Food composition tables and NutriSurvey software were used to assess mean amounts and contributions to recommended dietary allowances (RDAs) or adequate intake of energy, macronutrients, dietary fiber, vitamin C (VitC), iron, vitamin A (VitA), folate and vitamin B12 (VitB12). Results: On average, lunch sets provided roughly one third of RDA or adequate intake of energy, carbohydrates, fat and dietary fiber. Contribution to RDA of protein was high (46% RDA). The sets contained a high mean share of VitC (159% RDA), VitA (66% RDA), and folate (44% RDA), but were low in VitB12 (29% RDA) and iron (20% RDA). Conclusions: Overall, lunches satisfied recommendations of caloric content and macronutrient composition. Sets on average contained a beneficial amount of VitC, VitA and folate. Adjustments are needed for a higher iron content. Alternative iron-rich foods are expected to be better suited, compared to increasing portions of costly meat/fish components. Lunch provision at Cambodian garment factories holds the potential to improve food security of workers, approximately at costs of <1 USD/person/day at large scale. Data on quantitative total dietary intake as well as physical activity among workers are needed to further optimize the concept of staff canteens.

## 1. Introduction

Cambodia’s export-oriented garment industry has grown steadily over the past two decades, representing the mainstay of the country’s economy and accounting for 80% of total merchandise exports [1,2,3]. By midyear 2016, about 600 registered garment factories were operating in Cambodia, employing 610,000 workers [1]. Most factories manage low-value-added activities (“cut, make and trim”) and depend on imported fabrics and machinery, as well as on technical and supervisory personnel from abroad [2,3]. Factories are usually owned by foreign investors and are located in and around the suburbs of Phnom Penh, the capital of Cambodia [2,3]. The vast majority of garment workers are female (87%), mostly young women who migrate from low-income rural households [1,4]. Many have a poor school education, limiting their work options to agriculture and factory labor [4,5]. In 2016, the minimum wage for garment workers in Cambodia was set to 140 USD per month [1]. In addition to the minimum salary, workers greatly rely on bonuses, allowances and overtime work [4,6]. A large part of the earnings made while working in the factories are sent to family members which has an extensive anti-poverty effect there [4,5,6,7].

Concerns about the nutritional status of Cambodian garment workers were raised years ago [7]. It has been concluded that a proper diet in terms of quantity and quality is likely to be out of reach for this population group [4,6,7]. Malnutrition among workers has become a sensitive topic, as it has been linked to the mass faintings frequently reported in the factories [4]. The average daily amount of money spent by workers on food (~1.5 USD) has been described as insufficient to ensure an adequate diet [4]. Thrift measures also involve workers skipping meals [4,7]. In 2013, based on a small cross-sectional survey, NGOs reported a prevalence of 36% underweight among female workers [4]. A recent study conducted by the ILO in several Cambodian factories found 14% of workers to be underweight and 45% to be anemic [8]. Finally, the authors of this study reported 31% underweight, 27% anemia and a high prevalence of poor iron status from a factory-based baseline survey among young and nulliparous female garment workers [5].

Malnutrition (underweight, anemia and/or micronutrient deficiencies) among women in reproductive age is associated with impaired cognition, reduced work capacity and higher susceptibility to infections [9,10,11]. During gestation, it is associated with increased maternal morbidity and mortality, low birth weight, premature delivery and increased fetal and neonatal deaths [9,10,11].

Nutritional anemia is induced by diets that lack sufficient amounts of essential micronutrients, such as iron, vitamin A (VitA), vitamin B12 (VitB12) or folate, to meet the need for hemoglobin and red blood cell synthesis [9,12]. Non-nutrition factors for anemia are especially hemoglobinopathies, menstrual blood loss, and parasite infestations [9,13,14].

The establishment of staff canteens in Cambodian garment factories has been proposed as a suitable intervention to improve the nutritional and health status of workers, to reduce absenteeism, and to increase productivity [15]. Beyond that, meal provision showed positive effects on dietary diversity, on food security, and on lowering the percentage of employees who have taken loans for food purchases [8]. Still, most of the factories do not hold a canteen (or even an eating area) with the costs being the most critical factor [15]. At present, there is no national legislation obliging factory owners to operate canteens or to provide meals in any other way. Furthermore, national guidelines on meal provision in garment factories do not exist.

In spite of the publication of recent studies from Cambodia touching upon this subject [8,15], detailed information on exemplary meals/menus, their nutritive value and contribution towards recommended dietary allowances (RDAs), as well as their associated costs, are scarce or still missing. However, this information is essential to empower all stakeholders along the Cambodian garment sector to make informed choices on the setup and operation of staff canteens. The current paper reports on the exemplary lunch provision approach within the LUPROGAR study (Lunch Provision in Garment Factories), a factory-based randomized controlled trial, whose primary goal is to determine the impact of daily lunch provision through a staff canteen on the nutritional status (anthropometry and micronutrient status) of female garment workers in Cambodia. Based on food sample data collected during the trial’s lunch provision (non-systematic convenience sampling), the main objective of the present study is to determine the nutritive value (energy, macronutrients, dietary fiber and micronutrients) of twelve low-price model lunch sets considering the actual portion sizes.

## 2. Materials and Methods

### 2.1. Study Setting

The LUPROGAR trial was implemented during 2015 at Apsara Garment Co. Ltd., an export-oriented garment factory located in the suburban commune Chom Chau in Cambodia’s capital Phnom Penh, about 10 km west of the city center. The factory employed some 1300 workers. The majority were young unmarried women from low-income rural households. Conditions of employment were assumed to be comparable with overall working conditions in the garment sector. The factory operated on six workdays per week and was selected purposely since the management was showing interest to collaborate in this research.

**Figure 1:**
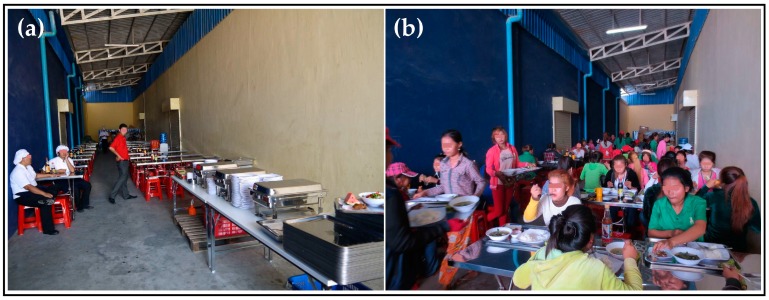
(**a**) Setup of the temporary canteen in a roofed area at factory site; and (**b**) dining area during lunch break (Depicted individuals were anonymized).

Following enrolment and baseline data collection in April 2015 [5], 223 female workers (<31 years old, non-pregnant and nulliparous) were randomly allocated in equal shares into an intervention arm (six months of free lunch provision during workdays) and a control arm (equal monetary compensation at the end of the study). The factory was previously not operating a staff canteen. A temporary canteen (including serving counter, dining area and crockery collecting station, about 100 seats capacity) was installed specifically for the LUPROGAR study. For this purpose, the management provided a roofed outdoor area (around 150 m^2^) at the factory site (see Figure 1). All needed materials and furnishing were locally purchased at markets and specialized shops (see Table 1). Total costs per seat amounted to 28 USD.

### 2.2. Lunch Provision

**Figure 2:**
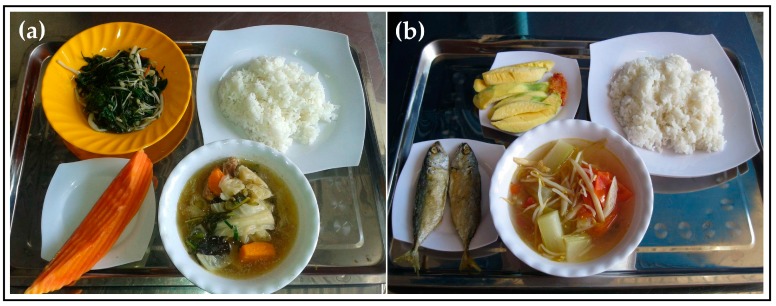
Examples of lunch sets (composed of stir-fry, soup, side item and fruit dessert) served at a garment factory in Phnom Penh, Cambodia: (**a**) stir-fried spinach (*Cha phti*), vegetable soup with pork (*Snau chab chay*), cooked rice and ripe papaya (Set B2); and (**b**) fried fish (*Trey chien*), Vietnamese vegetable soup (*Machu yuan*), cooked rice and mango (Set A5).

LUPROGAR aimed to serve adequate full lunch sets at reasonable costs (about 1 USD/person/day) in collaboration with Hagar Catering and Facilities Management Ltd., an established canteen service provider from Phnom Penh, Cambodia. First, lunch sets were drafted to be composed of a stir-fry dish, a soup dish, a side item (cooked rice), and a fruit dessert. The aim was to provide approximately 700 kcal/set, about one third of RDA for non-pregnant women aged 19–30 years old [16], in line with foreign guidelines on the caloric value of lunch provision through canteens [17]. A biweekly menu, including twelve model lunch sets, was then outlined in consultation with the caterer (see Table 2). Focus was laid on acceptable Cambodian dishes, on using local foods and on ensuring dietary diversity, by providing cereals, various vegetables, animal source foods (meat or fish), and fresh fruits on a daily basis. After one month of lunch provision, the menu was slightly adjusted according to preferences expressed by workers in a short menu preference questionnaire. Examples of lunch sets are illustrated in Figure 2.

After the setup of the canteen, lunch provision on workdays was carried out by the caterer for the duration of six months (from May until October 2015). Dishes were daily prepared according to consistent recipes at a commercial kitchen located in Phnom Penh’s city center and delivered within 1 h to the factory site. In soup dishes, the amount of non-fortified cooking oil per serving was targeted to be ~10 g. Stir-fry and soup dishes were reheated just before serving. The staff was instructed to serve constant portion sizes. At the canteen, workers had free access to drinking water and locally used condiments (non-fortified soy/fish sauce and fresh red chili).

The total net price of lunch sets, stipulated by the caterer, amounted to 1.15 USD/person/day and was determined by agreed serving sizes of 50 g animal source foods (0.50 USD), 150 g of vegetables (0.35 USD), 250 g of cooked rice (0.15 USD) and 100 g of fresh fruits (0.15 USD) (see Table 3). Animal source foods belong to the most expensive foods in Cambodia [15,18], explaining the relatively high price for the meat/fish component. The price per lunch set also reflects the relatively small order of about 100 lunch sets/day, as a discount is offered by the caterer with an increasing number ordered. In addition, the above price included delivery, food distribution, and cleaning services on a daily basis, as well as insurance covering eventual costs for treatment in the case of a foodborne disease.

### 2.3. Nutritive Value of Lunch Sets

On 50 days within the course of the six-month lunch provision (about 35% of total trial duration), single or multiple samples among two to all four dish types were taken directly from the service counter (non-systematic convenience sampling, at any arbitrary time of the actual food distribution) and subsequently weighed using a commercial electronic kitchen scale (SensorDisc SF-400, 500 g × 0.1 g, Huiding Hardware, Dongyang, China). Multiple samples per day were generally taken from fruit desserts, from single component dishes (stir-fry dishes such as fried fish, fried chicken wings and omelet) and from separately prepared fish portions in composite dishes, which were convenient to scale. The total minimum numbers of samples taken for each individual dish type were *n* = 5 for stir-fry dishes, *n* = 5 for soup dishes, *n* = 34 for the side item and *n* = 25 for fruit desserts (see Table A1 and Table A2 in Appendix A for exact number of samples). In composite dishes, all measurable ingredients were weighed individually. The amount of broth, if present, was determined by thoroughly decanting samples through a sieve.

Weight data were double entered into Excel spreadsheets and of each ingredient in every individual dish the mean and corresponding standard deviation were calculated (Excel 2013, Microsoft Corp., Redmond, Washington, WA, USA). Mean weights of the daily side item and of the six single fruit desserts are based on the overall number of samples taken in both menu weeks, A and B. Ingredients with a mean weight of ≤1.5 g were not considered in further analysis. Due to non-edible parts (skin or shell and seeds), a 30% weight loss was taken into account for two fruit desserts (banana and longan fruit) before further evaluation (based on the mean percentage of non-edible parts determined by weighing among 10 servings of each). All other fruit desserts were served peeled or peeled and seedless. The proportion of ingredients in the omelet dish was roughly estimated to be 80% egg and 20% climbing wattle (shoots of *Acacia pennata*, a shrub-like plant native to South/Southeast Asia).

Mean weights were used to estimate the nutritive value of all twelve lunch sets via NutriSurvey software (Version 29 October 2007, SEAMEO-TROPMED RCCN, University of Jakarta, Indonesia). For this purpose, food composition data for all 57 individual ingredients (data generally available as fresh/raw condition) were inserted into the NutriSurvey database, mainly from Cambodian and ASEAN food composition tables [19,20]. Vietnamese and USDA food composition tables were used for 10 ingredients which were not listed in the Cambodian nor ASEAN database [21,22]. Composition data for pitaya (dragon fruit) were found elsewhere [23]. Due to the limited data among the food composition tables, the nutritive value of lunch sets could only be estimated for energy, protein, fat, carbohydrates, dietary fiber, vitamin C (VitC), iron, VitA, and VitB12. Folate was included as well, although information on folate in the Asian databases was missing for 15 ingredients. Where possible, missing data on the folate content of these ingredients were completed with USDA data [22]. Table 4 shows the corresponding RDAs to calculate each lunch set’s contribution towards the RDAs among non-pregnant women aged 19–30 years old.

### 2.4. Ethics

The LUPROGAR trial was approved by the Institutional Review Board of the Faculty of Medicine at Justus Liebig University, Giessen, Germany (14 November 2014) and the National Ethics Committee for Health Research at the Ministry of Health, Phnom Penh, Cambodia (29 December 2014). Written informed consent was collected from all study participants prior to enrolment by signature or fingerprint. Both ethical committees approved the consent format prior to data collection. The study was registered at the German Clinical Trials Register (9 January 2015, Identifier: DRKS00007666).

## 3. Results

### 3.1. Amount of Food Groups/Ingredients in Lunch Sets

Table A1 and Table A2 (see Appendix A) illustrate the amounts by weight of individual ingredients for each lunch set provided during menu weeks A and B, respectively.

The mean weight of the meat/fish component in sets ranged from 64 ± 17 g (stir-fry dish in Set B3) to 15 ± 8 g (soup dish in Set A6), which was distinctly less than the agreed serving size of 50 g. However, in most of the sets, the mean weight of the meat/fish component was about 50 +/− 10 g. 

The total amount of vegetables provided with each lunch set (not separately shown here, based on the sum of mean weights of vegetables in both main dishes) varied considerably from 270 g (Set B2) to 93 g (Set A3). In general, more than the agreed serving size of 150 g vegetables was served. The highest amounts (>200 g) were found in lunch sets in which dark green leafy vegetables (DGLVs) or mixed vegetables were served as stir-fry dish (Sets A2, A4, A6 and B2). Moreover, in some soup dishes (Sets A1 and B4), as a common practice in Cambodia, considerable amounts of fresh DGLVs were added at serving (indicated as raw).

Broth (meat/fish based) was a main ingredient of soup dishes, with mean weights between 231 ± 31 g (Set A6) and 165 ± 25 g (Set A5). Further ingredients in main dishes included considerable amounts of egg (Set A3), fruits like pineapple and mango (Sets A5, A6, B1 and B3), sweet potato (Sets B1 and B5), soybeans (Set B1) and mushrooms (Sets B2 and B6). The mean amount of cooked rice was 279 ± 30 g, slightly higher than the agreed serving size of 250 g. Portion sizes of the fruit desserts ranged from 153 ± 38 g (papaya in Sets A2 and B2) to 82 ± 12 g (mango in Sets A5 and B5). Nonetheless, the agreed serving size of 100 g of fresh fruit was reached in four out of six desserts.

### 3.2. Nutritive Value of Lunch Sets: Calories, Macronutrients and Dietary Fiber

**Figure 3:**
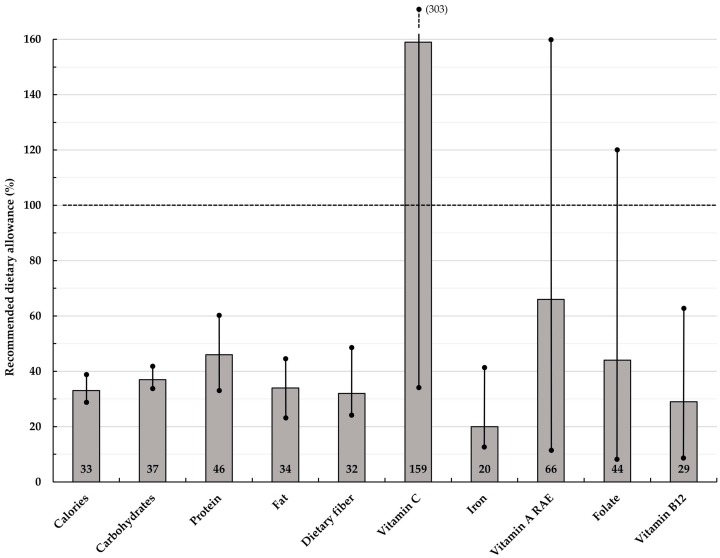
Mean contribution of lunch sets towards RDAs among non-pregnant women aged 19–30 years old. Lines within bars illustrate the range; RAE: Retinol activity equivalent.

The nutrient contents calculated for each lunch set are summarized in Table 5. In addition, Figure 3 shows the overall mean and range of the percentage contribution towards RDAs for all twelve sets. Compared to the micronutrients considered, the mean amounts of calories, macronutrients and dietary fiber remained rather constant and showed a lower variability.

Total energy content of the sets ranged from 591 kcal (Set A6, 28% of RDA) to 793 kcal (Set B1, 38% of RDA). In contrast to menu Week A, slightly higher values (730–793 kcal) were determined in four out of six sets from menu Week B. The side item (~280 g of cooked rice) provided the largest share of calories, roughly 360 kcal/set. On average, lunch sets provided 697 kcal (33% of RDA), with ~61% of calories derived from carbohydrates, ~23% from fats and ~13% from proteins (data not shown here). The amount of carbohydrates (100–112 g) was consistent among the majority of lunch sets (equaling 34–39% of RDA). Similar to the caloric value, the bulk of carbohydrates was provided with the portion of cooked rice (~82 g). Set B1 provided a slightly higher amount of carbohydrates (123 g, 42% of RDA), which was related to the amount of sweet potato used in the corresponding soup dish. Mean amount of carbohydrates among all sets was 107 g (37% of RDA).

The quantity of protein varied from 16 g (Set A6, 32% of RDA) to 30 g (Set B2, 60% of RDA). The low amount of protein in Set A6 was related to the relatively small amount of meat served with the corresponding soup dish. Still, most of the lunch sets contained 22–26 g of protein, which equaled 44–52% of recommended daily protein intake. In general, main protein sources were meat and fish. The mean amount among all sets was 23 g (46% of RDA). The fat content ranged from 12 g (Set A6, 23% of RDA) to 24 g (Set B4, 45% of RDA). The mean amount of fat was 18 g (34% of RDA). Slightly higher amounts (≥20 g) were noted in sets where fried fish (Sets A1, A5, B1), fried egg (Set A3) or fried meat (Set B4) were served as stir-fry dish. Lower amounts (<15 g) were calculated for sets including DGLVs (Sets A2, A4, B6) or mixed vegetables (Set A6) as stir-fry.

Lunch sets contained 6 g (Sets A3, A5, B4 and B6, 24% of RDA) to 12 g (Set B2, 48% of RDA) of dietary fiber, whereby DGLVs, fruits and rice were the main sources. On average, sets contained 8 g (32% of RDA) of fiber.

### 3.3. Nutritive Value of Lunch Sets: VitC, Iron, VitA, Folate and VitB12

Total VitC content varied considerably from 24 mg (Set A3, 34% of RDA) to 212 mg (Set B2, 303% of RDA). The richest sources were fruits (e.g., papaya and longan fruit) and vegetables (e.g., DGLVs). The average amount of VitC was 111 mg, which equaled 159% of recommended daily intake. The amount of iron in lunch sets ranged from 4 mg (Sets A1, A3, A5 and B4, 14% of RDA) to 12 mg (Set B2, 41% of RDA). DGLVs, some fruits, rice and meat/fish were the primary iron sources. The average amount was 6 mg, equaling only 20% of RDA.

The VitA content ranged greatly from 61 µg (Set B1, 12% of RDA) to 799 µg RAE (Set B2, 160% of RDA). Lunch sets including DGLVs such as morning glory (Sets A2, A4 and B3) choy sum (Set A4), spinach (Sets A6, B2, B5), or Chinese kale (Set B6) as main ingredients in stir-fry or soup dishes, were estimated to provide highest amounts of VitA (68–160% of RDA). Mean amount among sets was 331 µg RAE (66% of RDA).

The estimated amount of folate varied from 29 µg (Set A1, 7% of RDA) to 477 µg (Set B2, 120% of RDA). DGLVs and fruits were the main sources. Average amount among lunch sets was 175 µg, equaling 44% of RDA. The VitB12 content ranged from 0.2 µg (Set A6, 8% of RDA) to 1.5 µg (Set A5, 63% of RDA). Mean amount was 0.7 µg (29% of RDA). Lunch sets containing fish (Sets A1, A2, A5, B3, B5 and B6) were calculated to provide ≥0.7 µg of VitB12, compared to sets including pork or chicken meat (Sets A3, A4, A6, B2 and B4), providing lower amounts of VitB12.

## 4. Discussion

The present paper provides information on the caloric, macronutrient and micronutrient content of twelve low-price model lunch sets served to female garment workers in Cambodia. The assessment of the nutritive value is based on actual serving sizes determined during a six-month period in collaboration with an established commercial caterer. Food samples for analysis were obtained directly from the serving counter.

The caloric content of lunch sets accounted for about one-third of the recommended daily energy intake on average (697 of 2115 kcal/day), matching well the initially targeted goal for caloric value. It was estimated that ~61% of calories were provided by carbohydrates, ~23% by fat and ~13% by protein (data not shown here), which corresponds with FAO/WHO recommendations for sources of energy intake [27]. On the other hand, slightly different contributions, namely 50% from carbohydrates, 30% from fat and 20% from protein, have been proposed as an optimal macronutrient distribution [17]. Given the high prevalence of workers who showed a body-mass-index of less than 18.5 kg/m^2^ at LUPROGAR’s baseline survey [5], it remains unresolved if these average amount of 700 kcal per lunch is suitable to improve the nutritional status of workers affected by underweight. Quantitative data on the total daily dietary intake of Cambodian garment workers are still missing, both in general and in relation to their working conditions. However, those data are needed to evaluate the energy intake during lunch provision for dietary adequacy. Moreover, the RDA of 2115 kcal/day [16] might underestimate energy requirements among workers exposed to heavy work load and/or overtime work [25,26,27].

As for the caloric value of lunch sets, the estimated mean amount of carbohydrates (107 g), fat (18 g) and dietary fiber (8 g), each equaled roughly one-third of RDA (adequate intake with regard to dietary fiber). Contribution towards the RDA of protein was slightly higher (46%, 23 g of protein on average), which is regarded as beneficial, since protein intake among workers might be limited throughout the day due to the relatively high price of most animal source foods in Cambodia [15,18]. In summary, the amounts of macronutrients and dietary fiber provided by the exemplary sets seem to be balanced and sufficient. However, they would need to be adjusted if a higher caloric content of lunch sets is required. The limited extent of the accessible local food composition tables did not allow further characterization with respect to protein quality and composition of carbohydrates and fatty acids [19,20,21]. Nonetheless, total amounts of sugars (mainly derived from fruits) and saturated fatty acids (mainly derived from cooking oil and flesh meat) are considered to be within an acceptable range.

Compared to the macronutrients, the contribution of lunch sets towards RDAs of selected micronutrients showed a higher variability. Sets were estimated to provide a relatively high amount of VitC, on average 159% of RDA (111 mg), although values among single sets ranged substantially (34–303% of RDA). Intakes above current VitC RDAs have shown lowering effects on, for example, hypertension, endothelial dysfunction and chronic inflammation, independent risk factors for cardiovascular diseases and some cancers [28]. On the other hand, since composition data for many ingredients were only available as in fresh/raw condition, the estimations might likely overestimate total VitC content (in particular for vegetables and fruits in cooked or fried dishes). Despite the obvious lowering effect of boiling and frying [29], it is assumed that a sufficient amount of VitC is provided with lunch sets.

Average total iron content among sets was relatively low (6 mg) and equaled only 20% of RDA (assuming low bioavailability of 10%), which might not be adequate. Hence, it is doubted that the iron status of female garment workers could be improved. About two-third of LUPROGAR’s participants showed iron deficiency or a marginal iron status at baseline, but this information was not available at the time of the lunch sets planning. At least, a pajrt of the high prevalence of anemia is attributable to iron deficiency [5]. Most of the dietary iron was provided as less bioavailable nonheme iron, mainly derived from vegetables (especially iron-rich DGLVs, e.g., morning glory, spinach, and Chinese kale), fruits, and rice. On the other hand, VitC, present in adequate amounts, enhances nonheme iron absorption, but effects on iron absorption might be less pronounced in a complete diet including various dietary inhibitors [30,31]. Chicken and pork meat, as well as the various fish species, served in small portion sizes of ~50 g due to the relatively high price, have a low iron content (0.5–1.7 mg/100 g edible portion) [19,20,21]. Although iron bioavailability is higher from animal source foods [32,33,34], increasing the portion size of these foods would significantly increase the costs per meal. Other strategies to increase the iron content among the proposed lunch sets would be to include blood curd and liver (e.g., from chicken and pork) as common heme iron-rich food ingredients (up to 15 mg/100 g) in Southeast Asia [32]. They could be easily incorporated into single dishes and small amounts would already significantly increase the iron content of the lunch sets. Another food-based approach could be the incorporation of a locally available and traditionally used small fish, Mekong flying barb (*Esomus longimanus*) with a high total iron content of ~11 mg/100 g [35,36]. An alternative strategy could be the provision of iron-fortified fish/soy sauce or the use of iron-fortified rice [37,38]. However, assuming an average consumption of 5 g of fish/soy sauce during lunch, the utilization of iron-fortified sauces would only result in a modest increase in total iron content of 1.5 mg/lunch set (at fortification of ~300 mg iron/kg [37]). Moreover, alongside a national fortification program, undesired levels of nitrogen and salt content among iron-fortified sauces have been recently reported from Cambodia [37]. In addition, multi-micronutrient fortified rice (containing 7–11 mg iron/100 g uncooked rice) has been associated with increased risk of hookworm infections and showed only limited impact on improving the iron status of Cambodian school children [39,40].

The model lunch sets contained a relatively high amount of RAE on average (331 µg, equaling 66% of RDA). Still, VitA content also varied strongly (61–799 µg RAE), depending on the presence and portion size of various vegetables (e.g., DGLVs) and/or fruits (e.g., papaya) rich in provitamin A carotenoids. Both are accessible at a relatively low price and are part of the traditional Cambodian diet [18]. This illustrates how the proposed low-priced lunch sets contribute to an adequate VitA intake. However, the range of absorption and bioconversion of provitamin A carotenoids to VitA from various vegetables (compared to preformed VitA from animal source foods) can be low for DGLVs and other vegetables [41]. On the other hand, cooking and heat processing often result in greater bioavailability [29,41]. Nevertheless, at LUPROGAR’s baseline survey, female garment workers were not affected by VitA deficiency, and only a low prevalence of marginal VitA status was observed (<10%) [5].

The estimated mean folate content among sets was 175 µg, corresponding to 44% of RDA, which seems an adequate amount on average. Yet, single values ranged from 29–477 µg (7–120% of RDA), whereby DGLVs and fruits were the main sources again. The low amount in two sets, namely A1 (29 µg) and B1 (43 µg), may be due to underestimation, as data on folate content was missing for the corresponding fruit dessert (dragon fruit). The same applies for Sets A6 and B6 (folate data missing for longan). Folate deficiency is prevalent among Cambodian women, and measures to increase folate/folic acid intake have been suggested for interventions targeting the high prevalence of anemia [42].

On average, the sets contained 0.7 µg of VitB12 (29% of RDA). This amount appears to be adequate, since VitB12 deficiency among female workers was not found at LUPROGAR’s baseline survey, nor in national representative data among women of reproductive age [5,42]. Variations in VitB12 content were noted for different types of animal source foods. In lunch sets including fish, more VitB12 was found than in lunch sets with chicken or pork meat. The low VitB12 content in Set A6 was mainly related to the low quantity of meat in the corresponding soup dish. The loss of VitB12 from fish by various cooking methods seems to be low (2–15%); however, VitB12 bioavailability might be slightly lower than from flesh meats [43]. Unlike various flesh meats, fish is an affordable and widely consumed food, even among the socioeconomically disadvantaged populations in Cambodia [18,35]. It is assumed that a regular consumption of fresh and processed fish, also at meals outside the working hours, ensures an adequate VitB12 intake.

### Limitations of the Study

Unfortunately, the recipes for the model lunch sets do not rest upon LUPROGAR’s baseline findings regarding the nutritional and/or micronutrient status of participants [5], nor on any other previously conducted gap-oriented assessment. Since most of the analyses of the participants’ initial micronutrient status had to be conducted abroad, the results were available months later. 

The food composition assessment was solely focused on the provision through lunch sets and did not account for actual food leftovers (which, by plain observation, were estimated as very low). This is a limitation of the study, as contributions to RDAs are estimated based on the assumption that workers consumed the full meal.

A major limitation affecting the accuracy of the estimated mean nutritive values of single and all lunch sets was the non-systematic convenience sampling during the lunch provision (ranging from *n* = 5 for some stir-fry/soup dishes to *n* = 52 for a fresh fruit dessert). The small sample size among stir-fry/soup dishes partly led to large standard deviations when assessing the weight of individual ingredients in lunch sets. A systematic and consistent sampling would have been favorable, but unfortunately it could not be implemented within the study. Nevertheless, it is assumed that overall conclusions regarding the nutritive value of lunch sets are valid and informative.

The local food composition tables (Cambodian, ASEAN and Vietnamese) had a limited extent in terms of analysis and number of food items [19,20,21]. For this reason, the USDA food composition table was used for some ingredients (<10 items) [22]. The estimation of the nutritive value was therefore restricted and no statements could be made for protein quality, for composition of carbohydrates and fatty acids, and for components known to inhibit iron uptake [33,34]. Many food composition data were only available for fresh/raw condition, blending out the effect of cooking methods on the nutrient content. Finally, missing folate data for a few foods certainly led to an underestimation for some lunch sets.

## 5. Conclusions

Given the nutritional situation of Cambodian garment workers, meal provision through staff canteens is expected to bear the potential to improve food security of workers, approximately at costs of less than 1 USD/person/day (at large-scale). The estimations made here regarding the nutritive value of low-price model lunch sets provide a basis to review ongoing or planned lunch provision. Further research should focus on collecting data on quantitative total dietary intake as well as physical activity with respect to demanding working conditions. With such data, the concept of lunch provision could be further optimized.

LUPROGAR’s exemplary lunch sets for female garment workers matched foreign recommendations regarding their contribution to RDAs of caloric content and macronutrient composition for sources of energy intake. However, the micronutrient content revealed a low iron content in lunch sets. Thus, strategies are needed to increase the provision of iron. It is assumed that the incorporation of alternative and affordable iron-rich food items would be better suited to significantly increase the iron content than just increasing the serving sizes of rather costly meat/fish components in lunch sets. On the other hand, it is considered that model lunch sets contained beneficial and adequate amounts of VitC, VitA, folate and VitB12, on average. Considering the year-round availability and relatively low price for various vegetables and fruits in Cambodia, it should be feasible to ensure a suitable content of VitC, VitA and folate in meals served to workers through staff canteens.

## Figures and Tables

**Table 1:** Costs for the setup of the canteen (circa 100 seats capacity) ^1,2^.

Materials and Furnishing	Costs (USD)
Dining area	
Tables and chairs	750
Crockery ^3^	600
Water dispenser	120
Fans	220
Serving counter	
Tables	250
Gas cooker and supplies	150
Chafing dishes	450
Crockery for food distribution	40
Fire extinguisher	20
Crockery collecting	
Tables	100
Collecting boxes	100
**Total**	2800 ^4^

^1^ Roofed outdoor area (around 150 m^2^) provided by the factory management; ^2^ No canteen kitchen at site, daily food delivery from local caterer; ^3^ Including plates, bowls, cups, cutlery and trays; ^4^ Costs per seat: 28 USD.

**Table 2:** Biweekly menu for lunch provision at a garment factory in Phnom Penh, Cambodia ^1,2^.

Lunch Sets ^3^	Monday	Tuesday	Wednesday	Thursday	Friday	Saturday
**Identifier**	**A1**	**A2**	**A3**	**A4**	**A5**	**A6**
Stir-fry dish (Week A)	Fried fish *(Trey chien)*	Stir-fried morning glory *(Cha trakuon)*	Omelet with climbing wattle ^4^ *(Chien pong tea saom)*	Stir-fried choy sum *(Cha spey chong keus)*	Fried fish *(Trey chien)*	Stir-fried mixed vegetables *(Cha bonlai krupmuk)*
Soup dish (Week A)	Vegetable soup with pumpkin *(Samlor brahoeur)*	Winter melon sour soup with fish *(Samlor machu trolach)*	Banana blossom soup with chicken *(Samlor machu trayung chek)*	Morning glory sour soup with pork *(Machu kreung trakuon saichruk)*	Vietnamese vegetable soup *(Machu yuan)*	Spinach soup with minced pork *(Samlor phti snau saichruk)*
Side item (Week A)	Rice, cooked	Rice, cooked	Rice, cooked	Rice, cooked	Rice, cooked	Rice, cooked
Dessert (Week A)	Dragon fruit	Papaya	Banana	Pineapple	Mango	Longan fruit
**Identifier**	**B1**	**B2**	**B3**	**B4**	**B5**	**B6**
Stir-fry dish (Week B)	Fried fish with ginger and soybeans *(Trey chien choun)*	Stir-fried spinach *(Cha phti)*	Fried fish with pickled mango *(Chien trey khlang hai nuong svay)*	Fried chicken wing *(Slap morn bompong)*	Stewed fish *(Trey chien chu em)*	Stir-fried Chinese kale *(Cha kana)*
Soup dish (Week B)	Spicy vegetable soup with sweet potatoes *(Samlor ktis)*	Vegetable soup with pork *(Snau chab chay)*	Morning glory sour soup *(Machu kreung)*	Vegetable soup with green papaya *(Samlor koko)*	Spinach fish soup *(Snau Phti)*	Fish sour soup with onions *(Snau chru trey)*
Side item (Week B)	Rice, cooked	Rice, cooked	Rice, cooked	Rice, cooked	Rice, cooked	Rice, cooked
Dessert (Week B)	Dragon fruit	Papaya	Banana	Pineapple	Mango	Longan fruit

^1^ Six workdays per week; ^2^ Cambodian main dish names in brackets; ^3^ Stir-fry, soup, side item and fruit dessert; ^4^ Climbing wattle (*Acacia pennata*) is a shrub-like plant native to South/Southeast Asia, the feathery shoots are used here as a common ingredient.

**Table 3:** Agreed serving sizes and costs of food groups in low-price model lunch sets served at a garment factory in Phnom Penh, Cambodia. ^1^

Food Groups	Serving Size (g)	Costs (USD)
Main dishes (stir-fry and soup)		
Meat or fish, cooked or fried	50	0.50
Vegetables, cooked or fried	150	0.35
Cooking oil	10	-
Side item		
Rice, cooked	250	0.15
Dessert		
Various fruits, raw	100	0.15
**Total**	560	1.15 ^2^

^1^ According to agreement with caterer at a daily provision of about 100 lunch sets/day; ^2^ Total price per lunch set, including service costs (delivery, food distribution and cleaning) and insurance, excluding 10% VAT.

**Table 4:** Recommended dietary allowances (RDAs) for energy, macronutrients, dietary fiber, VitC, iron, VitA, folate and VitB12 among non-pregnant women aged 19–30 years old.

Variables	RDA	Source
Energy	2115 kcal/day ^1^	[16]
Macronutrients		
Carbohydrates	291 g/day ^2^	[24]
Fat	53 g/day ^3^	[25]
Protein	50 g/day ^1,4^	[16]
Dietary fiber	25 g/day ^5^	[26]
Micronutrients		
Vitamin C	70 mg/day ^1^	[16]
Iron	29.4 mg/day ^1,6^	[16]
Vitamin A (RAE)	500 µg/day ^1^	[16]
Folate	400 µg/day ^1^	[16]
Vitamin B12	2.4 µg/day	[25]

^1^ Based on a body weight of 50 kg; ^2^ Based on providing 55% of total energy intake; ^3^ Based on providing 22.5% of total energy intake; ^4^ Adjusted for 80% protein quality; ^5^ Adequate intake; ^6^ Adjusted for 10% bioavailability; RAE: Retinol activity equivalent.

**Table 5:** Estimated nutritive value of low-price model lunch sets provided at a garment factory in Phnom Penh, Cambodia (menu Weeks A and B) ^1,2^.

	Monday	Tuesday	Wednesday	Thursday	Friday	Saturday
**Identifier, Lunch Sets Week A**	**A1**	**A2**	**A3**	**A4**	**A5**	**A6**
Nutritive value						
Energy, kcal (% of RDA)	699 (33)	651 (31)	704 (33)	639 (30)	708 (34)	591 (28)
Carbohydrates, g (% of RDA)	101 (35)	100 (34)	103 (35)	104 (36)	103 (35)	102 (35)
Protein, g (% of RDA)	22 (44)	22 (44)	23 (46)	22 (44)	26 (52)	16 (32)
Fat, g (% of RDA)	21 (40)	14 (26)	20 (38)	13 (25)	20 (38)	12 (23)
Dietary fiber, g (% of RDA)	7 (28)	10 (40)	6 (24)	8 (32)	6 (24)	7 (28)
Vitamin C, mg (% of RDA)	47 (67)	167 (239)	24 (34)	206 (294)	75 (107)	106 (151)
Iron, mg (% of RDA)	4 (14)	8 (27)	4 (14)	9 (31)	4 (14)	7 (24)
Vitamin A (RAE), µg (% of RDA)	136 (27)	505 (101)	152 (30)	604 (121)	127 (25)	365 (73)
Folate, µg (% of RDA)	29 (7)	141 (35)	75 (19)	344 (86)	74 (19)	239 (60)
Vitamin B12, µg (% of RDA)	0.7 (29)	0.9 (38)	0.5 (21)	0.4 (17)	1.5 (63)	0.2 (8)
**Identifier, Lunch Sets Week B**	**B1**	**B2**	**B3**	**B4**	**B5**	**B6**
Nutritive value						
Energy, kcal (% of RDA)	793 (38)	749 (35)	730 (35)	765 (36)	686 (32)	646 (31)
Carbohydrates, g (% of RDA)	123 (42)	101 (35)	112 (39)	112 (39)	112 (39)	106 (36)
Protein, g (% of RDA)	23 (46)	30 (60)	24 (48)	24 (48)	24 (48)	24 (48)
Fat, g (% of RDA)	22 (42)	22 (42)	18 (34)	24 (45)	14 (26)	14 (26)
Dietary fiber, g (% of RDA)	10 (40)	12 (48)	8 (32)	6 (24)	8 (32)	6 (24)
Vitamin C, mg (% of RDA)	118 (169)	212 (303)	56 (80)	55 (79)	92 (131)	177 (253)
Iron, mg (% of RDA)	5 (17)	12 (41)	6 (20)	4 (14)	6 (20)	6 (20)
Vitamin A (RAE), µg (% of RDA)	61 (12)	799 (160)	340 (68)	142 (28)	372 (74)	370 (74)
Folate, µg (% of RDA)	43 (11)	477 (120)	126 (32)	97 (24)	210 (53)	245 (61)
Vitamin B12, µg (% of RDA)	0.6 (25)	0.4 (17)	1.2 (50)	0.3 (13)	1.2 (50)	1.0 (42)

^1^ Based on mean weights of ingredients in lunch sets; ^2^ In relation to RDAs as described in methods section; kcal: Kilocalories; RAE: Retinol activity equivalent; RDA: Recommended dietary allowance.

## Abbreviations

The following abbreviations are used in this manuscript:

USD	United States Dollar
NGO	Non-government organization
ILO	International Labour Organization
VitA	Vitamin A
VitB12	Vitamin B12
RDA	Recommended dietary allowance
LUPROGAR	Lunch Provision in Garment Factories
Kcal	Kilocalories
VAT	Value added tax
ASEAN	Association of Southeast Asian Nations
USDA	United States Department of Agriculture
VitC	Vitamin C
RAE	Retinol activity equivalent
DGLV	Dark green leafy vegetable
FAO	Food and Agriculture Organization of the United Nations
WHO	World Health Organization
SD	Standard deviation

## Appendix A

**Table A1:** Amount by weight of ingredients in low-price model lunch sets served at a garment factory in Phnom Penh, Cambodia (Week A) ^1,2^.

	Monday	Tuesday	Wednesday	Thursday	Friday	Saturday
**Identifier**	**A1**	**A2**	**A3**	**A4**	**A5**	**A6**
Stir-fry dish, Week A	**Fried fish**	**Stir-fried morning glory**	**Omelet with climbing wattle**	**Stir-fried choy sum**	**Fried fish**	**Stir-fried mixed vegetables**
Samples weighed	*n* = 22	*n* = 5	*n* = 20	*n* = 5	*n* = 24	*n* = 5
Ingredients (mean weight ± SD)	Snail eating barb fish, whole, fried (44 ± 12 g)	Morning glory, fried (119 ± 24 g) Vegetable broth (33 ± 24 g)	Egg omelet, fried (48 ± 13 g) ^3^ Climbing wattle, fried (12 ± 3 g) ^3^	Choy sum, fried (138 ± 20 g) Vegetable broth (76 ± 10 g)	Short mackerel fish, whole, fried (61 ± 11 g)	Tomato, fried (48 ± 11 g) Vegetable broth (46 ± 10 g) Onion, fried (33 ± 7 g) Cucumber, fried (25 ± 3 g) Pineapple, fried (15 ± 13 g) Chinese kale, fried (7 ± 3 g)
Soup dish, Week A	**Vegetable soup with pumpkin**	**Winter melon sour soup with fish**	**Banana blossom soup with chicken**	**Morning glory sour soup with pork**	**Vietnamese vegetable soup**	**Spinach soup with minced pork**
Samples weighed	*n* = 8	*n* = 8	*n* = 8	*n* = 6	*n* = 5	*n* = 5
Ingredients (mean weight ± SD)	Meat broth (185 ± 17 g) Sponge gourd, cooked (65 ± 32 g) Winter melon, cooked (51 ± 27 g) Pumpkin, cooked (31 ± 22 g) Ivy gourd leaves, raw (24 ± 7 g) Vegetable oil (10 g) ^4^ Oyster mushroom, cooked (3 ± 3 g)	Fish broth (176 ± 30 g) Winter melon, cooked (134 ± 29 g) Giant snake head fish steak, cooked (38 ± 9 g) Vegetable oil (10 g) ^4^ Rice paddy herb, raw (9 ± 6 g) Red chili, raw (5 ± 3 g)	Meat broth (225 ± 28 g) Banana blossom, cooked (69 ± 26 g) Chicken meat, cooked (33 ± 12 g) Vegetable oil (10 g) ^4^ Long coriander, raw (6 ± 7 g) Thai basil, raw (4 ± 6 g) Red chili, raw (3 ± 2 g)	Meat broth (172 ± 38 g) Morning glory, cooked (117 ± 23 g) Pork meat, cooked (31 ± 8 g) Vegetable oil (10 g) ^4^	Meat broth (165 ± 25 g) Tomato, cooked (69 ± 31 g) Winter melon, cooked (64 ± 8 g) Pineapple, cooked (13 ± 17 g) Vegetable oil (10 g) ^4^ Lotus root, cooked (7 ± 6 g) Rice paddy herb, raw (7 ± 5 g) Soybean sprouts, cooked (6 ± 7 g) Long coriander, raw (3 ± 5 g)	Meat broth (231 ± 31 g) Spinach, cooked (103 ± 13 g) Pork meat, minced, cooked (15 ± 8 g) Vegetable oil (10 g) ^4^
Side item	**Rice**	**Rice**	**Rice**	**Rice**	**Rice**	**Rice**
Samples weighed	*n* = 34 ^5^					
Ingredient (mean weight ± SD)	Rice, cooked (279 ± 30 g)					
Dessert	**Fresh fruit**	**Fresh fruit**	**Fresh fruit**	**Fresh fruit**	**Fresh fruit**	**Fresh fruit**
Samples weighed	*n* = 42 ^5^	*n* = 38 ^5^	*n* = 52 ^5^	*n* = 37 ^5^	*n* = 25 ^5^	*n* = 25 ^5^
Ingredient (mean weight ± SD)	Dragon fruit, raw (88 ± 15 g)	Papaya, ripe, raw (153 ± 38 g)	Banana, raw (102 ± 19 g) ^6^	Pineapple, raw (102 ± 33 g)	Mango, unripe, raw (82 ± 12 g)	Longan, raw(99 ± 7 g) ^6^

^1^ Each lunch set composed of stir-fry dish, soup dish, side item and fruit dessert; ^2^ Mean weights of ingredients used for the estimation of nutritive value of lunch sets; ^3^ Estimated amount of weight (omelet with 80% egg, 20% climbing wattle); ^4^ Estimated amount of weight according to agreement with caterer; ^5^ Overall number of samples taken in both menu weeks (Weeks A and B); ^6^ In further analysis adjusted for 30% weight loss due to non-edible parts (skin or shell and seeds), all other fruit desserts were served peeled or peeled and seedless; SD: Standard deviation.

**Table A2:** Amount by weight of ingredients in low-price model lunch sets served at a garment factory in Phnom Penh, Cambodia (Week B). ^1,2^

	Monday	Tuesday	Wednesday	Thursday	Friday	Saturday
**Identifier**	**B1**	**B2**	**B3**	**B4**	**B5**	**B6**
Stir-fry dish, Week B	**Fried fish with ginger and soybeans**	**Stir-fried spinach**	**Fried fish with pickled mango**	**Fried chicken wing**	**Stewed fish**	**Stir-fried Chinese kale**
Samples weighed	*n* = 5	*n* = 5	*n* = 5	*n* = 15	*n* = 5	*n* = 5
Ingredients (mean weight ± SD)	Snail eating barb fish, whole, fried (43 ± 19 g) ^3^ Onion, fried (28 ± 12 g) Ginger, fried (20 ± 4 g) Soybean, fermented, fried (16 ± 4 g)	Spinach, fried (136 ± 16 g) Vegetable broth (27 ± 28 g) Red chili, fried (2 ± 2 g)	Climbing perch fish, whole, fried (64 ± 17 g) ^3^ Mango, unripe, raw (54 ± 36 g) Tomato, raw (10 ± 10 g) Red chili, raw (3 ± 3 g)	Chicken wing, fried (61 ± 11 g)	Tomato, cooked (75 ± 25 g) Giant snake head fish, steak, cooked (48 ± 14 g) ^3^ Fish broth (44 ± 31 g)	Chinese kale, fried (115 ± 7 g) Vegetable broth (45 ± 13 g) Red chili, fried (2 ± 2 g)
Soup dish, Week B	**Spicy vegetable soup with sweet potatoes**	**Vegetable soup with pork**	**Morning glory sour soup**	**Vegetable soup with green papaya**	**Spinach fish soup**	**Fish sour soup with onions**
Samples weighed	*n* = 7	*n* = 6	*n* = 7	*n* = 7	*n* = 5	*n* = 5
Ingredients (mean weight ± SD)	Meat broth (175 ± 22 g) Sweet potato, white, cooked (79 ± 43 g) Green bell pepper, cooked (33 ± 15 g) Red bell pepper, cooked (26 ± 22 g) Pineapple, cooked (22 ± 11 g) Vegetable oil (10 g) ^4^	Meat broth (182 ± 33 g) Chinese cabbage, cooked (42 ± 40 g) Chinese kale, cooked (37 ± 18 g) Winter melon, cooked (28 ± 25 g) Pork meat, cooked (26 ± 18 g) Carrot, cooked (22 ± 15 g) Fried pork skin, cooked (20 ± 13 g) Wood ear mushroom, cooked (20 ± 16 g) Vegetable oil (10 g) ^4^ Spring onion, cooked (3 ± 3 g)	Meat broth (215 ± 35 g) Morning glory, cooked (121 ± 20 g) Vegetable oil (10 g) ^4^	Meat broth (200 ± 29 g) Papaya, unripe, cooked (78 ± 34 g) Pumpkin, cooked (23 ± 13 g) Jackfruit, unripe, cooked (23 ± 23 g) Ivy gourd leaves, raw (21 ± 7 g) Eggplant, cooked (16 ± 14 g) Vegetable oil (10 g) ^4^ Long beans, cooked (7 ± 5 g) Moringa leaves, raw (6 ± 6 g)	Fish broth (181 ± 32 g) Spinach, cooked (70 ± 26 g) Sweet potato, white, cooked (50 ± 57 g) Vegetable oil (10 g) ^4^ Giant snake head fish, steak, cooked (5 ± 6 g)	Fish broth (207 ± 25 g) Giant snake head fish, steak, cooked (44 ± 14 g) ^3^ Onion, cooked (31 ± 13 g) Oyster mushroom, cooked (25 ± 16 g) Vegetable oil (10 g) ^4^ Long coriander, raw (3 ± 3 g) Thai basil, raw (2 ± 2 g) Red chili, raw (2 ± 1 g)
Side item	**Rice**	**Rice**	**Rice**	**Rice**	**Rice**	**Rice**
Samples weighed	*n* = 34 ^5^					
Ingredient (mean weight ± SD)	Rice, cooked (279 ± 30 g)					
Dessert	**Fresh fruit**	**Fresh fruit**	**Fresh fruit**	**Fresh fruit**	**Fresh fruit**	**Fresh fruit**
Samples weighed	*n* = 42 ^5^	*n* = 38 ^5^	*n* = 52 ^5^	*n* = 37 ^5^	*n* = 25 ^5^	*n* = 25 ^5^
Ingredient (mean weight ± SD)	Dragon fruit, raw (88 ± 15 g)	Papaya, ripe, raw (153 ± 38 g)	Banana, raw (102 ± 19 g) ^6^	Pineapple, raw (102 ± 33 g)	Mango, unripe, raw (82 ± 12 g)	Longan, raw (99 ± 7 g) ^6^

^1^ Each lunch set composed of stir-fry dish, soup dish, side item and fruit dessert; ^2^ Mean weights of ingredients used for the estimation of nutritive value of lunch sets; ^3^ Number of samples of fish component, *n* = 20; ^4^ Estimated amount of weight according to agreement with caterer; ^5^ Overall number of samples taken in both menu weeks (Weeks A and B); ^6^ In further analysis adjusted for 30% weight loss due to non-edible parts (skin or shell and seeds), all other fruit desserts were served peeled or peeled and seedless; SD: Standard deviation.
